# EconoGNN: A graph neural network framework for temporal economic resilience insights

**DOI:** 10.1371/journal.pone.0343683

**Published:** 2026-04-22

**Authors:** Marcus Araujo, Francisco Rodrigues, Elaine Sousa

**Affiliations:** University of São Paulo (USP), São Carlos, Brazil; National University of Defense Technology, CHINA

## Abstract

Global economic shocks such as the 2008 financial crisis or recent trade escalations between the United States and China have exposed the complexity of interdependent economies and the need for systemic, multi-agent analysis. However, most regional economic resilience (RER) studies remain limited by localized datasets, inconsistent definitions, and static modeling approaches, restricting their ability to generalize insights across space and time. We introduce EconoGNN, a Graph Neural Network framework that integrates complexity theory, economic modeling, and machine learning to predict and explain regional economic resilience across 183 countries over 25 years. By combining over 81 million trade records (UN COMTRADE) and 500,000 macroeconomic observations (Penn World Table), and adopting an official resilience metric from the World Bank, our approach enables reproducible and interpretable global-scale analysis. EconoGNN achieves F1-scores of 0.750 with the temporal GNN architecture GConvGRU, AUC-ROC of 0.792, and PR-AUC of 0.757, demonstrating robust performance across different recovery threshold settings (τ = 0.90–1.00) with F1-scores ranging from 0.730 to 0.771, and yielding statistically significant improvements (p-value ≤0.05) over baselines. GNNExplainer validation confirms explanation reliability (Fidelity+ = 0.827, Characterization = 0.913), enabling country-customized interpretability of resilience drivers. Moreover the EconoGNN framework integrates key structural and welfare indicators to model both national and cross-border economic interactions, reducing omitted-variable bias and implicitly accounting for political, institutional, and cultural differences.

## 1. Introduction

Global geopolitics is becoming increasingly complex, with growing interdependencies between countries and financial systems in multiple layers. Among all the issues related to economic crises, two questions interest us: “Why do some regions recover quickly from economic shocks while others struggle for years?” and “Can advances in machine learning help uncover meaningful patterns within financial networks to address these disparities?”

Over the past decades, researchers have sought to answer these critical questions by analyzing lessons learned from global crises [1]. While advancements in Machine Learning (ML) have accelerated rapidly, there remains significant untapped potential for computer science and statistics to contribute to understanding and addressing these issues. These efforts aim to prevent economic instability, protect social well-being, and promote environmental sustainability. The field of Regional Economic Resilience (RER) directly engages with these challenges by studying ***“what enables certain regions to resist, recover, and adapt to disruptions while others cannot”*** [2–33]. Researchers have characterized RER as *“dynamic, multifaceted, multi-dimensional, and multi-factor, which means it evolves over time, incorporates various factors and dimensions, and is influenced by a range of determinants.”* Despite these advances, a recent survey [1] highlights persistent methodological and experimental challenges in RER studies. A review of 168 works published between 2000 and 2022 found inconsistencies in the definitions of economic resilience, even within the same article. Furthermore, 80.95% of these studies rely on empirical approaches with localized datasets or restricted samples of observed metrics, such as employment, population, or income. Purely qualitative studies make up 12% of the research, and among those using quantitative methods, only half (a total of 77) employed statistical approaches. This analysis reveals the following issues and opportunities in RER literature:

**Methodological and experimental issues**, such as: A) defining and detecting economic shocks; B) deciding the time frame for resilience analysis; C) choosing appropriate resilience indicators; and D) developing techniques capable of performing experiments on massive global datasets.

**Scalability and robustness issues** caused by the scarcity of: A) Massive data availability and quality; B) Dynamic modeling; C) Causal inference; and D) Predictive analysis.

A promising and foundational work published by the World Bank [34] provides a rigorous mathematical framework for addressing **Methodological and Experimental issues**. Notable advancements in other scientific domains demonstrate the efficacy of techniques such as Complex Networks and Machine Learning in addressing multi-dimensional, multi-factor problems [35]. Recent breakthroughs in Graph Neural Networks (GNNs) and Temporal Graph Neural Networks (TGNNs) have demonstrated the capacity to model agents and their interactions over time, yielding promising results for highly dynamic datasets [36–38]. However, state-of-the-art TGNN and GNN models remain underexplored in addressing interdisciplinary real-world problems, often lacking explainability in their findings. These limitations underscore the potential for developing a novel framework to address **Scalability and Robustness issues**.

Therefore, **this work introduces EconoGNN, a novel machine learning framework designed to predict and explain factors that drive Regional Economic Resilience across expansive global datasets**. Our framework tackles the mentioned opportunities in three phases: Data Integration and Definition; Feature Engineering; and Supervised Learning and Explainability (Table 1). Moreover, our approach incorporates: 1) a robust computational and statistical toolkit to ensure high-confidence results; 2) the World Bank’s rigorously defined mathematical models of economic resilience and shocks [34]; and 3) an extensive dataset comprising over 81 million records sourced from the United Nations World Commodities Trade and the World Penn Table, which integrates both macro and micro-economic indicators across various countries. Leveraging these resources, the EconoGNN aims to provide actionable insights and significantly improve the predictability of economic resilience on a global scale. EconoGNN contributes to the shock propagation literature by identifying structural asymmetries—labor- versus capital-driven propagation—within a temporal and cross-country framework, bridging traditional economic propagation models with graph-based learning approaches [39–41].

**Table 1:** EconoGNN Framework. Phases of the Related Methodological and Scalability Opportunities.

Phase	Objective	Related Opportunity
A) Data Integration and Definitions	Combine Massive Trade and Economic Indicators Datasets; Define resilience labels and levels from the parametrized economic resilience function; Create a labeled dataset to multiple supervised learning routines	Scarcity of Datasets; Defining and detecting shocks; Choosing Appropriate Resilience Indicator
B) Feature Engineering	Structure, enhance and summarize relevant features from countries and their neighborhoods across time, using causal statistical measures and Discrete-Time Dynamic Graph modeling	Dynamic Modeling; Causal Inference; Deciding the time frame for resilience analysis
C) Supervised Learning and Explainability	Explain real-world events through complex networks based on supervised learning and statistical explainability methods	Predictive Analysis; developing techniques capable of performing experiments on massive global datasets.

The following sections present: 1) Background on Regional Economic Resilience and Graph Neural Network Learning; 2) The EconoGNN framework; 3) Experimental results; and 4) Conclusion.

## 2. Background and Related Work

This section provides a concise examination of the foundational theories underlying Regional Economic Resilience (RER) and Graph Neural Networks Learning (GNN), which are essential for elucidating the proposed framework. In the discussion on RER, we offer an overview, incorporating the World Bank’s mathematical definitions. For GNN, we present the conceptual divisions and explore recent applications.

### Regional economic resilience

**Fig 1:**
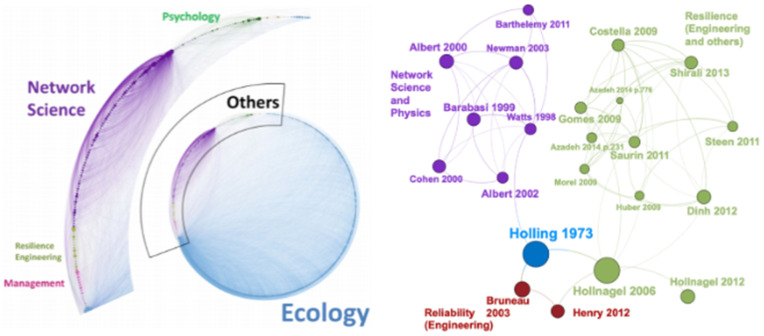
Resilience Literature. The last 25 years of works related to Resilience (left), and the most central citations (right). Adapted from [42].

The Literature on economic resilience is derived from ancient studies in other fields, such as Biology and Engineering. Moreover, a recent study [42] shows that different fields of science are highly connected, and indeed, even when works cite different conceptual backgrounds in Engineering, they are consequently referring to ecological foundation concepts. Fig 1 illustrates more than 20 years of publications around resilience, and besides the variety of works the citation network has shown to be extremely connected in their references and conceptual background to Network Science, Engineering, and, even more, to Ecology. Therefore, using Network Science, Engineering, or Ecological scientific advances to support Economic Resilience is not dystopic. These fields have a broad and deep understanding of resilience, including concepts and mathematical definitions, that are gradually being translated to economic purposes.

According to a recent survey [1], the Literature on RER clustered definitions in four groups: Engineering, Ecological, Evolutionary, and Transformative Resilience. For the present work, our framework is developed over what they call “Engineering Resilience” in reference to the World Bank Concept and Definitions [34]. Those concepts can be summarized in the following definitions:

**Engineering resilience:** The ability of regional economies to ‘bounce back’ from shocks to a pre-shock equilibrium [43–45].
**Ecological resilience:** The ability of regional economies to absorb shocks and maintain their current equilibrium by undergoing minimal structural and/or functional change [17,46,47].
**Evolutionary resilience:** The ability of regional economies to ‘bounce forward’ by adapting parts of their structures and functions in response to shocks [12,48–50].
**Transformative resilience:** The ability of regional economies to create new reconfigurations of their structures and functions in response to shocks [8,27,51,52].

Throughout our literature review, we’ve noticed a general approach based on local economic indicators and local (not global) datasets within restricted time frames, as well as a few formal mathematical definitions of resilience. The notable European economic resilience study [53,54] over the 2009 global crisis is an example of the most frequent analysis based on one specific economic indicator, and observability of spatiotemporal dispersion. The authors use the unemployment rate to measure economic resilience, restricting the perspective of resilience to a labor-oriented indicator. The study utilizes statistical databases from the European Union for the period from 2007 to 2011, focusing on the financial crisis that began between 2008 and 2009. Resilience measurement is based on the variation of the unemployment rate compared to the pre-crisis period (2007) and categorizes countries into four groups: resilient, recovered, unrecovered in decline, and unrecovered in ascent. Resilient countries are those that maintained their employment levels during the crisis. Recovered countries are those that experienced a drop in unemployment levels but managed to return to similar levels as before the crisis. The unrecovered countries in decline or ascent are those with lower employment rate levels with a trend of decrease or increase, respectively.

A recent contribution that advances the methodological landscape in Regional Economic Resilience (RER) modeling is FLEE-GNN [55], a graph-based learning system designed to predict agricultural resilience by regressing over supplier–customer dependency rates across U.S. states. The model applies Graph Neural Networks (GNNs) to capture topological patterns of inter-state food flows, utilizing a federated and edge-enhanced architecture that allows decentralized learning and preserves data locality. This framework leverages the U.S. Commodity Flow Survey (CFS), a complex and large-scale dataset, comprising relational trade information across multiple dimensions, with publicly available tables ranging from dozens to millions of records. These features position FLEE-GNN as a technically sophisticated attempt to integrate machine learning with structured economic interdependencies.

However, despite its architectural rigor and the adoption of advanced multi-agent based modeling (GNN), the study is limited in several critical dimensions. It focuses exclusively on two static years (2012 and 2017), lacks a formal definition of resilience aligned with institutional standards (e.g., World Bank), and is constrained to a single national context (USA) with no temporal dynamics or cross-country generalization. Furthermore, the resilience measure used is custom-built, based on dependency ratios, and not grounded in established productivity-based frameworks. These limitations, particularly in terms of temporal resolution, global scope, and theoretical grounding, open room for more comprehensive frameworks capable of generalizing across regions and over time, while also incorporating explainability, standardized resilience definitions, and global-scale data integration.

Another relevant contribution in the economic forecasting domain is DoomBot [56], an OECD initiative that applies machine learning techniques to improve recession forecasting. DoomBot uses probit models with quarterly data from 1980 onward to predict recessions over horizons up to two years for 20 OECD countries, relying primarily on financial indicators such as credit, house prices, share prices, and yield curve slopes. Unlike Trade-GNNs such as those proposed by Sellami et al. [57], which focus on edge-level regression tasks (predicting trade flow volumes), DoomBot performs node-level classification (predicting economic states), making it a methodologically comparable baseline to our approach. We include DoomBot in our experimental evaluation to assess the added value of global coverage, trade network topology, and resilience-focused metrics. Nonetheless, pure recession forecasting undermines resilience assessment, which requires measuring not only crisis onset but also recovery thresholds and capacity.

### World bank economic resilience measure to label crisis periods

To tackle the Replicability and Generalizability deficits that most RER works remain, we employ an official mathematical definition provided by a relevant Economic institution, The World Bank [34]. The authors not only produced generic definitions and amplified the potential of multi-country (local and global) analysis, but also increased the confidence level of future analysis due to the importance of the World Bank.

**Fig 2:**
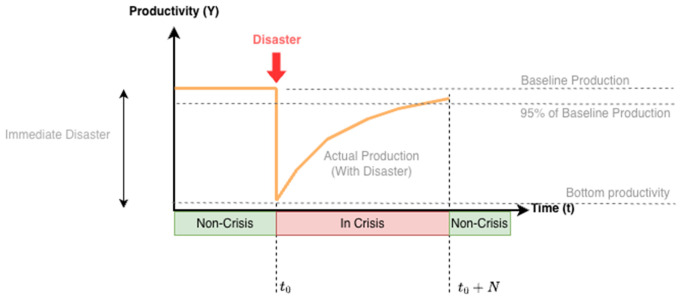
Resilience Labeling. Resilient Economics Recovery, assuming 95% threshold (Productivity-Based Approach). Adapted from [34].

The mathematical definition of economic resilience unified the semantic problematic and well-known nations’ economic indicators (e.g., Gross Domestic Product – GDP). Concisely, a country can be considered “In Crisis” when its productivity rate hasn’t reached the stable recovery threshold. For example, the recovery threshold could be considered to be 95% of the original productivity level prior to the most recent economic disaster. Otherwise, it can be considered “Non-Crisis,” i.e., during recovery or stability. Definitions 1, 2 and 3 represent our simplification on how it’s possible to define economic resilience labels and map the current productivity to them, given on a historical horizon.


$$\begin{array}{c} L_{𝒞}\in \left\{ \begin{array}{cc} \{\text{"Crisis"},\;\text{"Non-Crisis"}\}, & K=2\\ \{\text{"Crisis"},\text{"Recovery"},\text{"Stable"}\}, & K=3\\ \{L_{1},L_{2},L_{3},\ldots ,L_{K}\}, & K>3 \end{array}\right. \end{array}$$ (1)

$$𝒞(Y,t,ℋ,\tau )=1+\sum_{j=1}^{K-1}\;\left\{\frac{Y(t)}{\max_{k\in [t-ℋ,\;t-1]}Y(k)}>\tau_{j}\right\}$$ (2)

$$ℒ(Y,t,ℋ,\tau )=L_{\;𝒞(Y,t,ℋ,\tau )},$$ (3)

where *Y* represents the productivity level of a country (denoted in USD), τ is the percentual recovery threshold (e.g., 95%), ℋ is the temporal horizon to be analyzed (non-negative integer), and *t* represents the current instant (non-negative integer). Fig 2 illus*t*rates the financial damage (or disaster), immediate impact, and recovery of the country’s productivity.

Moreover, it’s possible to derive the accumulated impact. Equations 4 and 5 define the immediate disaster over productivity and the total loss, respectively.


$$\Delta Y(t)=\frac{1+\alpha }{\mu }r\Delta Ke^{-\frac{3(t-t_{0}^{-})}{N}}$$ (4)

$$\Delta Y=\int_{t_{0}^{-}}^{+\infty }\frac{1+\alpha }{\mu }r\Delta Ke^{-\frac{3(t-t_{0}^{-})}{N}}dt$$ (5)

Both equations are described in terms of the immediate loss of productivity $\Delta Y(t_{0}^{-})$ (measured in monetary units, e.g., USD) in the disaster instant ($t_{0}^{-}$: where productivity levels starts to get lower than the record, as defines e.q. 3), economic growth rate (1+α|α∈ℝ), the cost of the marginal capital -a real interest rate expressed on an annual basis (r|r∈ℝ)-, production loss (μ|μ∈ℝ∈[0,1]), and the capital value loss measured in monetary units (ΔK|ΔK∈ℝ). The annual recovery factor (3/*N*) is interpreted as “the characteristic time,” and its value is derived from the recovery rate in logarithmic trend (assuming τ= 95%), where exp(−3)≈0.05 and therefore $\Delta Y(t_{0}+N)=0.05\Delta Y(t_{0})$. It is also important to note that it can be adapted to other thresholds, and, if the productivity time series is relatively short, it’s always possible to set a pre-set productivity baseline to replace the empirical maxima (component $\max_{k\in [tℋ,\;t-1]}Y(k)$ of eq. 2).

### Countries economies represented as discrete time dynamic graph structure

Considering the World Bank’s economic resilience function to label countries over time, we can define a binary classification (Crisis, Non-Crisis) or multi-class classification (e.g., Crisis, Recovery and Stable). We also need a mathematical approach to integrate countries’ behaviours temporally. Multi-agent and temporal analysis are usually associated with complex mathematical, computational, and economic models. Among the most frequent models, Graphs (or Complex Networks) are robust and have reached solid results [58–62]. The graph structure is mathematically defined and can be static or dynamic, depending on the application problem. Temporal Graphs, represented in discrete or continuous timeframes, stand out for temporal applications. Our work considers Dynamic and Temporal Graphs in Discrete timeframes, also known as Discrete Time Dynamic Graph (DTDG).

**Fig 3:**
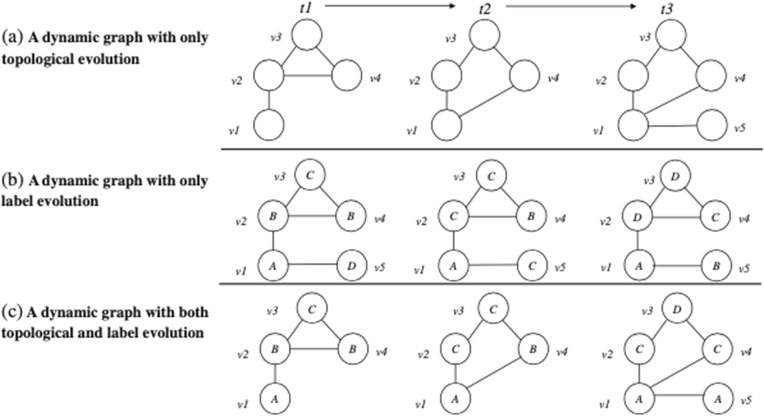
Discrete-Time Dynamic Graph Types. Node Classification in Discrete-Time Dynamic Graph Models. Adapted from [63].

The Computer Science Literature defines the DTDG temporal evolution in three perspectives (Fig 3): A) a dynamic graph with only topological evolution; B) a dynamic graph with only label evolution; and C) a dynamic graph with both topological and label evolution. The global economic resilience domain belongs to the third variant (C), i.e., a country (node) could be “Non-Crisis” or “In Crisis” (labels) in different moments of the period, and it could change its commercial partners (edges) over time.

A DTDG with topological and label evolution enables our framework to retrieve knowledge from global economic resilience variation (label evolution), and global trade crisis propagation (topological evolution).

### Graph neural networks supervised learning

**Fig 4:**
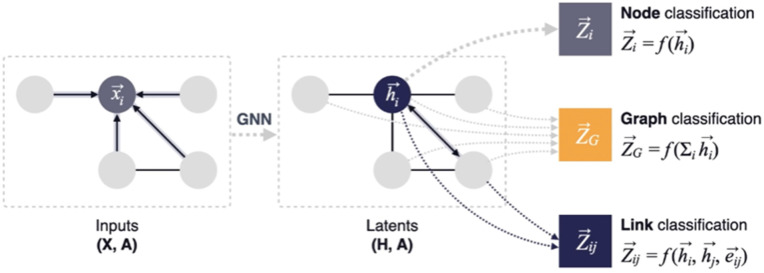
Node, Graph and Edge Classification. An overview of Graph Neural Networks tasks based on Graph Structure (Node Features *X* and Adjacency Matrices ***A*)**.

Graph Supervised Learning can be categorized into: Node Classification, Graph Classification, or Link Classification. **Node classification** is about predicting the label (*Z*_*i*_) of each node (*h*_*i*_) contained on graph set based on its features (*f*), i.e., $Z_{i}=f(h_{i})$. **Link prediction** is about predicting the likelihood (*Z*_*i*,*j*_) of two nodes ($h_{i},h_{j}$) connect based on their behavior and neighborhood (*e*_*i*,*j*_) similarity($Z_{i,j}=f(e_{i,j},h_{i},h_{j})$), and **graph classification** goal is to predict a label for an entire graphs based on its structure and node/edge attributes ($Z_{G}=f\left(\sum_{i}h_{i}\right)$) – Fig 4.

Regarding temporal graphs, two main approaches have been identified: **Snapshot-based models**, which handle temporal graphs as sequences of snapshots, and **Event-based models**, which process events like node or edge updates dynamically. Both model groups can be set up into different combinations of learning settings and tasks (node classification, link prediction, or graph classification).

Graph Neural Networks (GNNs) are designed to efficiently learn from graph structures [64] and have been extensively surveyed in recent comprehensive reviews [65]. In particular, Temporal Graph Neural Networks (TGNNs) are an evolution of this structure that considers temporal information in supervised learning from temporal graphs. Given the inherently dynamic nature of trade networks and the propagation of economic shocks over time, TGNNs are particularly well-suited to capture evolving dependencies across countries. Their ability to model asynchronous, high-frequency interactions directly addresses limitations of static models in capturing delayed or cascading resilience phenomena [66,67].

The transition from Graph Neural Networks (GNNs) to Temporal Graph Neural Networks (TGNNs) constitutes a paradigm shift that transcends the mere inclusion of temporal information in graph structures. While traditional GNNs operate on static graphs—where nodes and edges are fixed throughout the training process—TGNNs are explicitly designed to capture dynamic systems, where the topology and attributes of nodes and edges evolve over time [68]. This temporal dimension introduces unique challenges that reshape not only the data representation but also the model architecture, training procedures, and learning objectives. GNNs typically address node classification, graph classification, or link prediction in a single snapshot, whereas TGNNs extend these tasks to a predictive setting across time, such as forecasting node labels or link existence in future time steps [69,70]. Architecturally, TGNNs integrate sequential modeling components—such as memory modules, temporal attention mechanisms, or recurrent neural networks—to capture the causal influence and temporal dependencies between graph events [68,71]. Furthermore, TGNNs often require event-based processing and fine-grained temporal reasoning, distinguishing them from snapshot-based GNNs that treat time as a discrete series of graphs. These distinctions underscore that TGNNs are not a simple temporal extension of GNNs but a fundamentally different modeling framework, particularly suited for scenarios involving high-frequency, asynchronous, and time-sensitive interactions. Consequently, TGNNs represent a methodological advancement that bridges the gap between temporal modeling and graph-based learning, enabling more accurate and context-aware inference in dynamic systems [72].

GNN and TGNN learning strategies can also be divided into their mechanisms [64]: Convolutional, Attention or Autoencoder. **Convolutional Mechanisms** are the most common aggregation methods paradigm in graph analysis, using convolutional or pooling operations on graph structure to extract higher representation for each node and then used in node classification [73–75]. **Attentions Mechanisms** allow variant weights among neighbors, focusing on the most relevant part [76]. **Autoencoder Mechanisms** is usually applied to a low-dimensional embedding from large unlabeled training data (semi-supervised learning) [77–79].

Specifically to the purpose of the present work, **we focus on Node Classification, varying among Convolutional, Attention, and Autoencoder mechanisms**. We explore the following five methods to learn from global economic resilience:

**GraphSage:** A Graph Neural Network (GNN) architecture that performs learning on graphs, generating node representations based on their neighbors [75].
**GAT (Graph Attention Network):** A GNN that uses attention mechanisms to weigh the importance of each neighbor of a node, improving accuracy in heterogeneous graphs [80].
**GCN (Graph Convolutional Network):** A GNN model that applies convolutions on graphs to capture structural information and features from neighboring nodes [74].
**ChebNet:** A GNN that uses spectral convolutions based on Chebyshev polynomials, efficient for capturing local and global patterns in graphs [73].
**GIN (Graph Isomorphism Network):** A GNN that excels in differentiating complex graph structures, used for classification tasks and robust node representation [81].

Beyond static GNN architectures, we also evaluate Temporal Graph Neural Networks (TGNNs) that explicitly model sequential dependencies through recurrent or memory-based mechanisms:

**GConvGRU:** Combines Graph Convolutional layers with Gated Recurrent Units (GRU), enabling the model to capture both spatial graph structure and temporal dynamics through gated memory updates [82].
**GConvLSTM:** Integrates Graph Convolutions with Long Short-Term Memory (LSTM) cells, providing enhanced capacity for learning long-range temporal dependencies in dynamic graphs [82].
**TGCN (Temporal Graph Convolutional Network):** A hybrid architecture combining GCN with GRU for spatiotemporal forecasting, originally designed for traffic prediction but applicable to economic time series [82].
**DCRNN (Diffusion Convolutional Recurrent Neural Network):** Models spatial dependencies as a diffusion process on graphs while capturing temporal dynamics through sequence-to-sequence learning with scheduled sampling [82].
**EvolveGCN-O / EvolveGCN-H:** Adaptive architectures that evolve GCN parameters over time using recurrent networks (GRU or LSTM), allowing the model to adapt to structural changes in dynamic graphs without requiring node-level recurrence [68].

On applied research using Node Classification, varying among Convolutional, Atttention and Autoencoder mechanisms, we highlight three groups of related work on: Social Network Analysis, Human Mobility and Epidemic Modeling.

On Social Network analysis [60,61], authors applied node classification on social networks to help predict the roles or groups of individuals based on their connectivity and interactions over time.

On Human Mobility [59], authors applied TGNNs to classify nodes representing individuals based on their mobility patterns. These patterns would be later crucial for urban and traffic management.

On Epidemic Models [58,62], authors applied node classification to identify the likely inffections statuses of individuals based on their interactions and movements to leverage the entire known network during training.

Finally, among all related research, we couldn’t find any work that combines official economic measures, global public datasets, and robust supervised learning techniques to predict countries’ regional economic resilience. Therefore, we propose the EconoGNN Framework, aiming to explore those opportunities.

## 3. EconoGNN: A Graph neural network framework for temporal economic resilience insights

To move beyond traditional approaches, we propose an innovative framework that employs machine learning and global financial networks to uncover the deeper mechanisms driving economic resilience.

For the present work’s purpose, running a multi-country analysis involves considering domestic and international information to understand the conditions that lead to a country’s economic state. This perspective is materialized through a Discrete-Time Dynamic Graph (DTDG). Formally, we define the discrete-time dynamic graph at time *t* as:


$$G_{t}=(V_{t},E_{t},X_{t},ℒ(t))$$

where *V*_*t*_ is the set of nodes (countries) at time *t*, $E_{t}\subseteq V_{t}\times V_{t}$ is the set of directed edges representing trade relations between countries, $X_{t}\in {\mathbb{R}}^{|V_{t}|\times d}$ is the feature matrix containing *d*-dimensional economic attributes per country (e.g., capital stock, employment, human capital), and $\{0,1{\}}^{\left|V_{t}\right|}$ is the label vector indicating the resilience state of each country (0 for “In Crisis,” 1 for “Non-Crisis”).

Over the time horizon *T*, the dataset becomes a sequence of snapshots:


$$𝒢=\{G_{1},G_{2},...,G_{T}\}$$

capturing both topological evolution (in *E*_*t*_) and label evolution in line with the third category of DTDG, as discussed in the background session.

**Fig 5:**
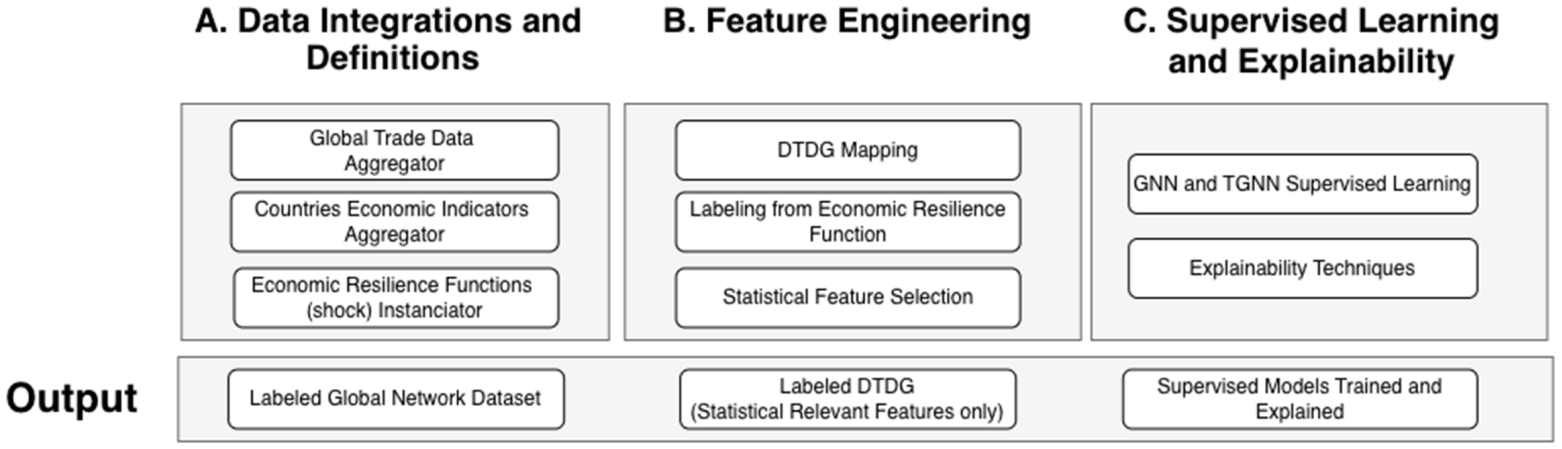
EconoGNN Framework: Steps and Outputs.

Considering the mentioned related works, issues, and opportunities, we present The EconoGNN framework (Fig 5) capable of unifying: 1. massive global datasets; 2. mathematical and official definitions of economic resilience; 3. a robust methodological graph supervised learning setup. Our methodology aims to solve RER mapped opportunities (Table 1) and is structured in 3 steps: A) Data Integrations and Definitions; B) Feature Engineering; C) Supervised Learning and Explainability.

In the **Data Integrations and Definitions** phase, the global trade and countries indicators aggregator is responsible for collecting and structuring data from official services (United Nations COMTRADE and Penn World Table). Then, using economic resilience functions, the global trade dataset contains temporal information of each economy, respective relationships, and a set of economic perspectives on resilience.

In the **Feature Engineering** phase, we build a discrete-time dynamic graph (DTDG), labeled from following the economic resilience definition, retrieve and reduce the information set to the most relevant features using statistical methods.

On **Supervised Learning and Explainability**, we run GNN supervised learning on a model competition template, so the best candidates can be explained using well-known explainability methods.

### Public economic datasets

To address the challenge of data scarcity, this study harnesses two exceptionally detailed and significant datasets: the Penn World Table (PWT) and the United Nations COMTRADE. The first is detailed in Table 2, which divides the dataset into 8 groups of variables, being them: Identifier variables; Real GDP, employment, and population levels; Current price GDP, capital, and TFP; National accounts-based variables; Exchange rates and GDP price levels; Data information variables; Shares in CGDPo; Price levels, expenditure categories, and capital.

**Table 2:** Penn World Table Data Dictionary. PWT version 10.

Variable Name	Variable Definition
countrycode	3-letter ISO country code
country	Country name
currency_unit	Currency unit
year	Year
rgdpe	Expenditure-side real GDP at chained PPPs (in mil. 2017US$)
rgdpo	Output-side real GDP at chained PPPs (in mil. 2017US$)
pop	Population (in millions)
emp	Number of persons engaged (in millions)
avh	Average annual hours worked by persons engaged
hc	Human capital index, based on years of schooling and returns to education; see Human capital in PWT9.
ccon	Real consumption of households and government, at current PPPs (in mil. 2017US$)
cda	Real domestic absorption, (real consumption plus investment), at current PPPs (in mil. 2017US$)
cgdpe	Expenditure-side real GDP at current PPPs (in mil. 2017US$)
cgdpo	Output-side real GDP at current PPPs (in mil. 2017US$)
cn	Capital stock at current PPPs (in mil. 2017US$)
ck	Capital services levels at current PPPs (USA = 1)
ctfp	TFP level at current PPPs (USA = 1)
cwtfp	Welfare-relevant TFP levels at current PPPs (USA = 1)
rgdpna	Real GDP at constant 2017 national prices (in mil. 2017US$)
rconna	Real consumption at constant 2017 national prices (in mil. 2017US$)
rdana	Real domestic absorption at constant 2017 national prices (in mil. 2017US$)
rnna	Capital stock at constant 2017 national prices (in mil. 2017US$)
rkna	Capital services at constant 2017 national prices (2017 = 1)
rtfpna	TFP at constant national prices (2017 = 1)
rwtfpna	Welfare-relevant TFP at constant national prices (2017 = 1)
labsh	Share of labour compensation in GDP at current national prices
irr	Real internal rate of return
xr	Exchange rate, national currency/USD (market+estimated)
pl_con	Price level of CCON (PPP/XR), price level of USA GDPo in 2017 = 1
pl_da	Price level of CDA (PPP/XR), price level of USA GDPo in 2017 = 1
pl_gdpo	Price level of CGDPo (PPP/XR), price level of USA GDPo in 2017 = 1
i_cig	0/1/2/3/4: relative price data for consumption, investment and government is extrapolated (0), benchmark (1), interpolated (2), ICP PPP timeseries: benchmark or interpolated (3) or ICP PPP timeseries: extrapolated (4)
i_xm	0/1/2: relative price data for exports and imports is extrapolated (0), benchmark (1) or interpolated (2)
i_xr	0/1: the exchange rate is market-based (0) or estimated (1)
i_outlier	0/1: the observation on pl_gdpe or pl_gdpo is not an outlier (0) or an outlier (1)
i_irr	0/1/2/3: the observation for irr is not an outlier (0), may be biased due to a low capital share (1), hit the lower bound of 1 percent (2), or is an outlier (3)
cor_exp	Correlation between expenditure shares of the country and the US (benchmark observations only)
statcap	Statistical capacity indicator (source: World Bank, developing countries only)
csh_c	Share of household consumption at current PPPs
csh_i	Share of gross capital formation at current PPPs
csh_g	Share of government consumption at current PPPs
csh_x	Share of merchandise exports at current PPPs
csh_m	Share of merchandise imports at current PPPs
csh_r	Share of residual trade and GDP statistical discrepancy at current PPPs
pl_c	Price level of household consumption, price level of USA GDPo in 2017 = 1
pl_i	Price level of capital formation, price level of USA GDPo in 2017 = 1
pl_g	Price level of government consumption, price level of USA GDPo in 2017 = 1
pl_x	Price level of exports, price level of USA GDPo in 2017 = 1
pl_m	Price level of imports, price level of USA GDPo in 2017 = 1
pl_n	Price level of the capital stock, price level of USA in 2017 = 1
pl_k	Price level of the capital services, price level of USA = 1

The Penn World Table (PWT) offers a comprehensive set of 47 GDP uncorrelated variables for analysis of global economic conditions for each country. It chronicles the economic annual behavior of 183 countries from 1950 to 2019 and comprises over 500,000 records.

**Fig 6:**
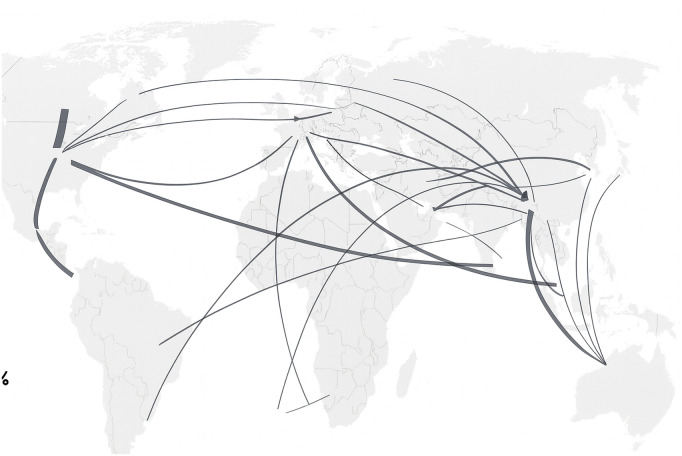
Global Financial Network. Global commercial network illustration, based on COMTRADE 2019 top financial flows.

The COMTRADE database is a comprehensive United Nations repository that captures the global financial network (Fig 6). It collects annual trade statistics delineated by product, originating country, and destination country, amassing over 1 billion records spanning annual time series from 1961 to 2022. Each record in this dataset includes details such as the traded commodity (classified according to the Harmonized System, HS), source and destination countries (indicated by their official abbreviations), mode of transportation (land, sea, or air), quantity, and financial value (expressed in millions of dollars).

## 4. Experimental results

This section is structured to reflect the EconoGNN pipeline, guiding the reader through each phase of our approach. We begin by detailing the Data Integration process, including key data volumes and DTDG outputs. Then, describing the feature engineering process, and, finally, exploring Shallow Supervised, Graph Neural Networks Learning and Explainability.

### Data integration and feature engineering

We’ve applied our framework over COMTRADE and PWT datasets, labeled by the World Bank Economic Resilience Function ℒ – eq 3-. Generating 58 DTDG snapshots of 183 countries, based on the overlaping periods of PWT and COMTRADE datasets (1966–2019). These 23 years have GDP levels for 100% of the globe, and the previous years have shown missing data at some level. For every country, the feature set contains over 50 domestic and centrality measures (betweenness and closeness) varying along temporal snapshots as commercial partnerships rearrange. The features of a country were also enhanced and increased by appending the features of its relevant neighbors.

**Fig 7:**
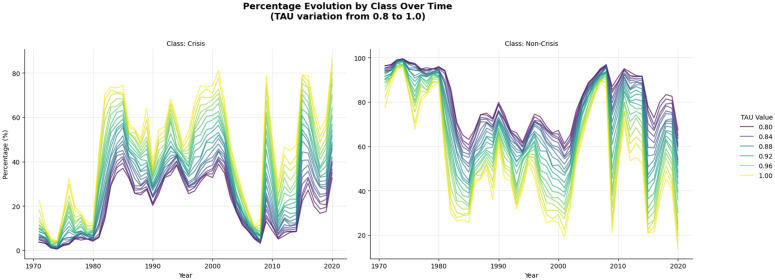
variation and impact on labeling balance. Varying τ from 0.8 to 1, the labeling process defined in equations 1, 2, and 3, generates less (τ→0) or more conservative (τ→1) recovery diagnoses. The number of countries with reported productivity varies until 1996, but after that, data is considerably more available for the globe.

It’s important to highlight that the recovery threshold τ was set to be the most conservative τ=1, to avoid misclassifications of “Non-Crisis,” and integrate one or combined shocks as “Crisis.” Fig 7 illustrates how the variation of this parameter can affect the distribution of labels.

Table 3 describes the DTDG snapshots’ behavior annually, counting the sum of commercial connections (Edges) in the current snapshot, average number of connections to each country (⟨k⟩), Network Profile [35], and proportion of countries in crisis. It’s interesting to highlight some changes across years: **1.** The number of edges more than doubled from 1996 to 2019 and peaked at 17,709 in 2017; **2.** The average number of commercial connections also increased significantly during this period and reached a stability of nearly 155 connections, but the intensity of those connections varied substantially; and **3.** the network lost the characteristics of a Small World Network in five moments, which shows a topological change.

**Table 3:** Discrete Time Dynamic Graph Statistics. Global network behavior along time illustrated by the DTDG metrics on total number of connections (edges), average number of connections to each node, network profile and % os nodes in crisis.

DTDG Snapshot – Year	Edges	⟨k⟩	Network Profile
1996	7,538	71.79	Small W. & Regular
1997	10,082	96.02	Small W. & Regular
1998	11,144	106.13	Regular Network
1999	12,047	114.19	Small W. & Regular
2000	14,096	128.15	Small W. & Regular
2001	14,655	133.23	Small W. & Regular
2002	15,236	138.51	Small W. & Regular
2003	15,516	141.05	Small W. & Regular
2004	15,904	144.58	Small W. & Regular
2005	16,076	146.15	Small W. & Regular
2006	16,534	149.63	Small W. & Regular
2007	16,974	153.61	Small W. & Regular
2008	17,181	155.48	Small W. & Regular
2009	17,157	155.27	Small W. & Regular
2010	17,365	156.44	Regular Network
2011	17,470	155.98	Regular Network
2012	17,582	156.98	Regular Network
2013	17,688	157.23	Small W. & Regular
2014	17,578	156.95	Small W. & Regular
2015	17,683	157.88	Small W. & Regular
2016	17,595	157.10	Small W. & Regular
2017	17,709	158.12	Small W. & Regular
2018	17,525	155.78	Regular Network
2019	16,061	143.40	Small W. & Regular

To guarantee maximal statistical confidence and computational efficiency, we’ve also investigated the linear predictive potential of variables, to prioritize experiments on high potentials and avoid data leakage on suspicious variables. This analysis is based on varying temporal windows length from 1 to 15, aggregating features, and predicting the next year. Table 4 compiles the Information Value (IV) of high potential variables, from which we can affirm that the population size of the current and previous year has shown significant participation in resilience prediction, but also employment (present and previous year), capital stock (present and previous year), the price level in household consumption (present and previous two years), capital stock in constant prices (present and previous year), the price level of domestic absorption (present and previous year), the price level of consumption (present and previous year), and, finally, capital services (present and previous year). The IV score eliminated GDP correlated and component variables (e.g., import and export levels).

**Table 4:** Variable Descriptions and IV Values. Variables with higher predictive model and non-suspicious or correlated relation to the target variable (avoiding data leakage).

Variable	Description	IV
pop	Population (pop)	0.45
T1_pop	Population, previous year (T1_pop)	0.45
emp	Employment (emp)	0.43
T1_emp	Employment, previous year (T1_emp)	0.43
T1_cn	Capital stock, previous year (T1_cn)	0.43
cn	Capital stock (cn)	0.43
pl_c	Price level, household consumption (pl_c)	0.41
T1_pl_c	Price level, household consumption, previous year (T1_pl_c)	0.41
T2_pl_m	Price level, imports, previous year (T2_pl_m)	0.40
T1_rnna	Capital stock, constant prices, previous year (T1_rnna)	0.39
rnna	Capital stock, constant prices (rnna)	0.39
T2_pl_x	Price level, exports, previous year (T2_pl_x)	0.37
pl_da	Price level of domestic absorption (pl_da)	0.35
T1_pl_da	Price level of domestic absorption, previous year (T1_pl_da)	0.35
pl_con	Price level of consumption (pl_con)	0.32
T1_pl_con	Price level of consumption, previous year (T1_pl_con)	0.32
T1_rkna	Capital services, constant prices, previous year (T1_rkna)	0.31
rkna	Capital services, constant prices (rkna)	0.31

### Supervised learning and explainability

Different from linear statistics metrics, supervised learning has the potential to find non-trivial associations between input and output datasets. To do so, we set up a grid search to develop a smart search on parameters capable of finding maximal assertion, then use supervised learning explainers to uncover determinant behaviors. In addition to classification metrics, we also applied the formal resilience function (eq. 5) to quantify real recovery trends for selected countries. This metric serves as a numerical proxy for economic bounce-back and allows validation of the model’s outputs from an official, policy-aligned standpoint.

For comparison effects, on Traditional Supervised Learning, we’ve varied different temporal windows from 1 to 15 past years of economic behavior to predict the next year in the economic state. We’ve also varied commercial import (*N*_*in*_), export (*N*_*out*_), and granger (*N*_*granger*_) causally associated partnerships, levels of top importers and exporters (top partners that sum up 0–100% of total flow), and specific parameters for each shallow method. This experiment generated stable results over 0.7 F1-Score metrics, with significant levels of confidence, illustrated in Table 5. From this, we can extract the superiority of our model’s performances against a simple coin toss (Dummy Classifier). Among shallow supervised classifiers, Histogram Gradient Boosting and Random Forest reached high statistical relevance (p-value ≤0.1). Histogram Gradient Boosting had better statistical confidence considering only the exporter’s information (0.698, and p-value ≤0.05), and Random Forest set with 100 estimators and 30 levels (depth) has a better absolute result (0.722). It’s important to highlight that all the winning models chose 12% as the threshold of top exports and imports participation to determine a minimal relationship level on input and output financial flows.

**Table 5:** Shallow Methods Performance. Best results on grid search using shallow supervised learning on F1-Score, average precision and recall (a: p-value ≤5%; b: p-value ≤10%).

Supervised Method	Approach	F1-Score	$\mu_{P}$	$\mu_{R}$
Random Forest (hight:30, Estimators:100)	${𝒩}_{in}(v_{i},0.12)$	**0.722(0.161)** ^*a*^	0.798	0.734
Histogram Gradient Boosting	${𝒩}_{in}(v_{i},0.12)$	**0.717(0.165)** ^*a*^	0.794	0.731
AdaBoostClassifier (Estimators:100)	${𝒩}_{out}(v_{i},0.12)$	0.605(0.169)^*b*^	0.677	0.628
Gaussian Naive Bayes	${𝒩}_{in}(v_{i},0.12)$	0.569(0.169)	0.628	0.600
Naive Bayes	${𝒩}_{out}(v_{i},0.12)$	0.556(0.176)	0.615	0.585
Dummy Classifier	Prior	0.375 (0.257)	0.310	0.515
Isolation Forest (Estimators:100)	${𝒩}_{in}(v_{i},0.12)$	0.256(0.206)^*b*^	0.242	0.289

For shallow supervised methods, SHAP scores [83] reveal that neighborhood information consistently dominates predictions, with trading partner identities and their capital services emerging as the most discriminative features. The top commercial partners -such as United States, Argentina, and China- collectively account for the largest SHAP contributions, alongside lagged capital services variables from these partners (SHAP ranging from 0.06 to 0.33). Notably, these feature importance rankings remain relatively fixed across countries, as shallow methods cannot capture node-specific topological dependencies. This limitation motivates our detailed interpretability analysis using GNNExplainer for Temporal GNNs, which provides country-customized explanations that vary according to each nation’s unique position in the trade network.

On Graph Neural Network Supervised Learning (GNN and TGNN), we’ve trained our models using 1 million epochs, and varied temporal windows from 1 to 15, hidden channels (32,64 and 128), number of layers (3,5,6,7,12), optimizers (SGD,ADAM, ADAMW, RMSPROP), learning rate (1e-3, 1e-5, 1e-9, 1e-12), dropout (0, 0.3, 0.5, 0.7), and weight decay (0 and 1e-2).

Results compiled in Table 6 demonstrate the clear superiority of Temporal Graph Neural Networks (TGNNs) over both static GNNs and external benchmarks. GConvGRU achieved the highest F1 score (0.750) with precision of 0.803 and recall of 0.755, using a configuration of 5 temporal windows, 64 hidden channels, 2 layers, Adam optimizer, learning rate of 1e-3, and dropout of 0.2. GConvLSTM (F1 = 0.740) and TGCN (F1 = 0.733) followed closely, all sharing the same optimal architecture—suggesting that the temporal modeling component, rather than the specific recurrent mechanism, drives performance gains. Notably, all three TGNNs substantially outperform DoomBot [56] (F1 = 0.647, Precision = 0.650, Recall = 0.660), the OECD’s machine learning approach for recession forecasting, demonstrating a + 16% improvement in F1-Score. The comparison with DoomBot is methodologically appropriate as both approaches perform node-level classification (country economic states), unlike Trade-GNNs that focus on edge regression tasks. Static GNN architectures achieved considerably lower performance, with GraphSAGE at F1 = 0.602, followed by ChebNet and GCN at 0.586.

**Table 6:** GNN and TGNN Performance. Comprehensive results including Temporal GNNs (TGNNs) and the DoomBot benchmark. W = temporal window, HC = hidden channels, #L = layers, Opt = optimizer, LR = learning rate, Drop = dropout, WD = weight decay (a: p-value ≤5%; b: p-value ≤10%).

Model Name	W	HC	#L	Opt	LR	Drop	WD	F1	$\mu_{P}$	$\mu_{R}$
GConvGRU	5	64	2	adam	1e-03	0.2	0	**0.750** ^*a*^	0.803	0.755
GConvLSTM	5	64	2	adam	1e-03	0.2	0	0.740^*a*^	0.802	0.747
TGCN	5	64	2	adam	1e-03	0.2	0	0.733^*a*^	0.793	0.739
DoomBot	–	–	–	–	–	–	–	0.647	0.650	0.660
GraphSAGE	9	64	12	rmsprop	1e-05	0.5	0	0.602^*b*^	0.639	0.650
ChebNet	11	128	3	rmsprop	1e-09	0.7	0.01	0.586^*b*^	0.627	0.650
GCN	11	128	5	adamw	1e-09	0.7	0	0.586^*b*^	0.577	0.670
GAT	9	128	6	adamw	1e-12	0.7	0	0.580^*b*^	0.610	0.651
GIN	11	128	6	rmsprop	1e-05	0.5	0	0.575^*b*^	0.217	0.392

Furthermore, Table 7 presents the threshold sensitivity analysis, showing that GConvGRU performance remains robust across different recovery threshold values (τ = 0.90, 0.95, 1.00), with F1-scores ranging from 0.730 to 0.771 and AUC-ROC consistently above 0.77. Notably, the model achieves the highest MCC (0.488) at τ = 1.00, while maintaining well-calibrated probability estimates across all thresholds (Brier Scores: 0.176–0.223). This validates our methodological choice and demonstrates that the model captures meaningful resilience patterns rather than being sensitive to arbitrary threshold selection.

**Table 7:** Threshold Sensitivity Analysis. Performance of GConvGRU across different recovery thresholds τ (highest MCC at τ = 1.00; low Std indicates robustness).

Metric	τ = 0.90	τ = 0.95	τ = 1.00	Std
F1-Score	0.771	0.730	0.750	0.017
Accuracy	0.785	0.744	0.755	0.017
Precision	0.796	0.771	0.803	0.013
Recall	0.785	0.744	0.755	0.017
MCC	0.416	0.420	0.488	0.033
AUC-ROC	0.772	0.778	0.792	0.008
PR-AUC	0.586	0.675	0.757	0.070
Brier Score	0.176	0.207	0.223	0.020

**Fig 8:**
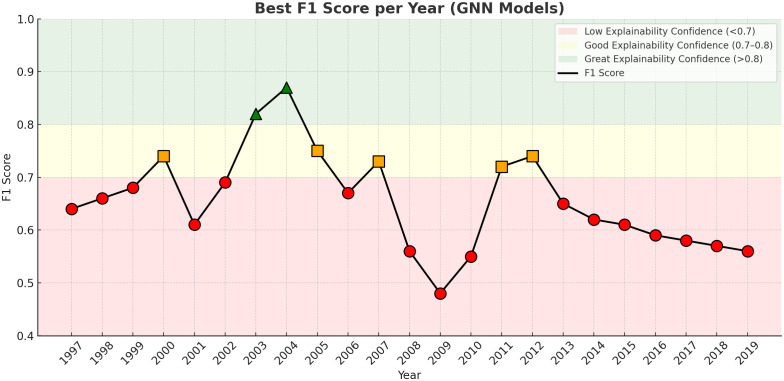
GNN Best Performance on F1 Score overall. The time series represent the best score overall gnn methods for the year, and highlight low, good and great results.

**Fig 9:**
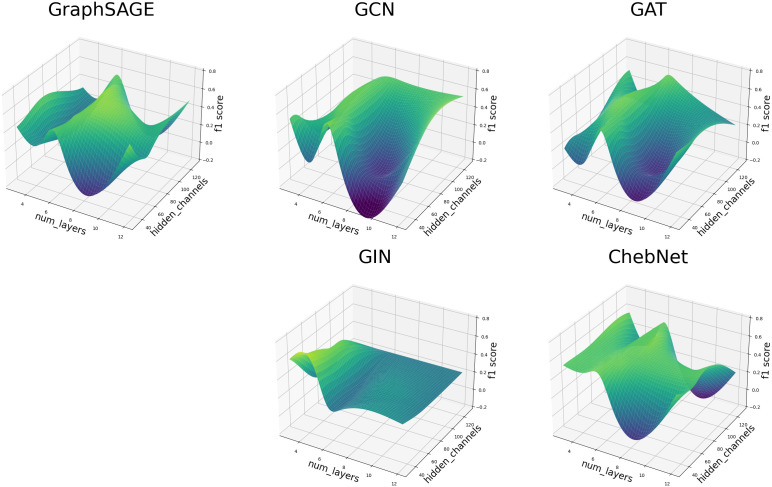
GNN Methods F1 Score Surfaces. Performance surfaces over hidden channels and the number of layers.

Fig 8 presents the best F1-Score achieved by GNN methods for each year, highlighting periods of low, good, and great explainability confidence. After a thorough investigation into the factors contributing to the uneven performance of GNN F1-Scores across years, we identified a highly sensitive hyperparameter landscape that leads to performance spikes, as illustrated in Fig 9. These results reveal a degree of stability in GraphSAGE, GAT, and ChebNet models when configured with six layers. However, all models exhibit substantial variance and multiple local maxima, underscoring the critical influence of precise hyperparameter tuning on their overall performance. We also highlight the importance of temporal information to the model performance and stability.

To understand the decision process of the best-performing model (GConvGRU), we applied GNNExplainer [84] across the entire study period. The analysis achieved high explanation quality: Fidelity+ of 0.827±0.378, indicating that in 82.7% of cases, removing the features and edges identified by GNNExplainer significantly altered the model’s prediction, confirming that explanations capture genuinely important factors. The near-zero Fidelity- suggests that predictions depend on information distributed throughout the trade network, consistent with the systemic nature of economic crises. The mean Characterization score of 0.913 indicates high overall explanation quality. Filtering for high-confidence predictions (≥80%), which cover 60.3% of cases spanning all 24 years, we observed superior explanation quality: Fidelity+ of 0.856 (+3.5% vs. overall) and Characterization of 0.927 (+1.5% vs. overall).

Table 8 presents the ten most influential economic indicators for predicting regional economic resilience across all the models adjusted to each country. The prevalence of lagged variables (T1_, T2_ prefixes) confirms that the model effectively utilizes temporal patterns. International trade price indices emerge as the most discriminative attributes, with two-year lagged export and import price levels occupying the top positions—corroborating the theoretical importance of terms-of-trade shocks as triggers of economic crises [56].

**Table 8:** Most Influential Economic Indicators. Feature importance from GNNExplainer for GConvGRU model.

Rank	Feature	Description	%
1	T2_pl_x	Export Price Level (t-2)	19.8
2	emp	Employment	19.0
3	T2_pl_m	Import Price Level (t-2)	18.7
4	rkna	Real Capital Stock	18.3
5	T1_pl_c	Consumption Price Level (t-1)	18.0
6	T1_pl_da	Domestic Absorption Price (t-1)	18.0
7	T1_rkna	Real Capital Stock (t-1)	17.3
8	pl_c	Consumption Price Level	17.1
9	T1_pop	Population (t-1)	17.1
10	pl_da	Domestic Absorption Price	17.0

Table 9 presents the most influential trading partners identified through edge structure analysis. The United States emerged as the most influential partner (22.6%), reflecting its central role in global trade networks. China (16.5%) and Japan (13.8%) represent dominant Asian economies, while Germany (13.5%) and United Kingdom (12.5%) anchor European influence. The presence of Russia, India, and United Arab Emirates among the top ten indicates the growing importance of emerging economies and energy producers.

**Table 9:** Most Influential Trading Partners. Edge importance from GNNExplainer for GConvGRU model.

Rank	Country	%
1	USA	22.6
2	CHN	16.5
3	JPN	13.8
4	DEU	13.5
5	GBR	12.5
6	ITA	10.1
7	BEL	7.6
8	RUS	7.3
9	IND	7.2
10	ARE	6.7

The analysis of high-influence subgraphs (normalized values above 0.3) identified country clusters characterized by geographic proximity and historical-linguistic ties, as shown in Table 10. These clusters could reflect structures of trade dependency shaped by geography, colonial history, and regional integration agreements.

**Table 10:** Trade Dependency Clusters. Main clusters identified by GNNExplainer edge analysis.

Cluster	Anchors	Influenced Countries	Characteristic
South America	BRA, ARG	PRY, URY, BOL, CHL, PER, COL	Mercosur + Andean
Lusophone	PRT, BRA	AGO, MOZ, CPV, GNB, TLS	Portuguese ex-colonies
Hispanic-American	ESP, MEX	GTM, SLV, HND, NIC, CRI, PAN	Central America
Anglophone African	GBR, ZAF	NGA, KEN, GHA, UGA, TZA	African Commonwealth
Francophone African	FRA, BEL	SEN, CIV, CMR, MLI, BFA, NER	CFA Franc Zone
Central Eurasia	RUS	KAZ, UZB, BLR, UKR, GEO, ARM	Ex-Soviet republics
Southeast Asia	CHN, JPN	THA, VNM, MYS, IDN, PHL	ASEAN + China
Persian Gulf	ARE, SAU	BHR, KWT, OMN, QAT	Cooperation Council

Importantly, no single feature or country accounts for more than 23% of cases, indicating that there is no universal predictor of economic resilience. This highlights that TGNN-based prediction provides country-customized explanations, detecting the relative weights specific to each nation’s position in the trade network—a capability that shallow methods fundamentally lack. This stability analysis, aligned with best practices for interpretable machine learning [85], ensures that our findings are robust rather than artifacts of specific model configurations.

## 5. Limitations and future work

The present study offers a reproducible and interpretable framework for modeling cross-country economic resilience through graph-based learning. Nevertheless, several limitations delineate opportunities for methodological and empirical advancement.

First, although the performance analysis indicates that the proposed models outperform random and dummy classifiers by a statistically significant margin (p-value ≤0.05), and the best-performing model GConvGRU achieves F1 = 0.750 with AUC-ROC of 0.792, there remains room for improvement. This reflects the intrinsic non-stationarity of macroeconomic data and the structural complexity of resilience phenomena. The evaluation metrics employed in this study—including Precision, Recall, F1-Score, MCC, AUC-ROC, PR-AUC, and Brier Score—follow established guidelines for imbalanced classification tasks [84,86]. The achieved PR-AUC of 0.757 and Brier Score of 0.223 indicate well-calibrated probability estimates, addressing concerns about model reliability in imbalanced settings [87]. Furthermore, moving beyond fixed-window approaches to rolling-origin evaluation schemes would strengthen temporal validity.

Second, the selection of canonical graph neural network architectures was a deliberate methodological choice. Rather than serving as exploratory placeholders, these models represent the **theoretically stable and widely reproducible baselines** across the main paradigms of graph representation learning (spectral, spatial, attention-based, and aggregation-based). This decision ensures transparency and comparability, aligning with open-source implementations in PyTorch Geometric and supporting methodological robustness in line with recent surveys [66,67]. The combination of spectral (GCN, ChebNet), spatial (GraphSAGE), and attention (GAT) mechanisms allows for capturing multi-scale topological and temporal dependencies without introducing architectural bias. We will expand the tests for other GNNs such as GraphSAGE extensions, GraphSMOTE, ProGNN, and StableGNN to improve both predictive stability and structural interpretability once larger and event-level datasets become available. Besides the node classification approach, we see the possibility of expanding the discrete label representation to a continuous one, enabling comparisons with gravity baselines, a modern trade-GNN [57], and interaction with attention proxies [88].

Third, it is important to clarify that the **interpretability achieved by EconoGNN is descriptive rather than causal**. Feature importance estimates are intended to provide transparent insights into which economic signals contribute most strongly to resilience classifications, not to establish causal inference. Nonetheless, this descriptive interpretability offers meaningful policy-relevant insights.

Fourth, data granularity remains an open frontier. The current setup relies primarily on annual COMTRADE and World Bank indicators, which limit temporal resolution. Our findings suggest that GNN performance improves with longer and denser time series, emphasizing the need to extend **data granularity and temporal coverage**. This will enable the construction of exposure/centrality measures from the learned model and test them in structural propagation regressions with proper robustness. Future iterations of EconoGNN will integrate modeling strategies capable of handling heterogeneous and multi-resolution time series, reconciling sources that cannot be published at sub-annual frequencies due to GDPR and confidentiality constraints.

Finally, future developments will focus on strengthening the framework’s computational efficiency, real-time scalability, and adherence to ethical and open-science standards. These advances will position **EconoGNN** as a robust, transparent, and adaptive platform for studying economic resilience under evolving global conditions.

## 6. Conclusion

This study presents **EconoGNN**, a robust and scalable framework designed to analyze and predict regional economic resilience. By addressing both methodological and scalability issues in the literature, EconoGNN represents a significant advancement in integrating global datasets, rigorously defined economic resilience measures, and advanced machine learning techniques. EconoGNN was designed under strict ethical and transparency principles, using only public and aggregated data, and setting the foundation for future extensions that incorporate privacy-preserving mechanisms and responsible AI standards. Moreover, the framework is designed so that users can modify the shock or resilience definitions to match specific economic perspectives or experimental setups. This flexibility is core to EconoGNN’s objective is to be a general-purpose resilience analysis tool.

The framework’s adoption of Graph Neural Networks (GNNs) enables a nuanced understanding of how domestic factors and international trade dynamics interact over time. Our findings emphasize the importance of combining country-specific features with network-level insights to uncover the patterns that influence economic stability and crisis recovery. More specifically, our work collaborates with:

**Predictive Power and Robustness**: Temporal Graph Neural Networks (TGNNs) achieved the highest predictive performance, with GConvGRU reaching F1 = 0.750, Precision = 0.803, Recall = 0.755, AUC-ROC = 0.792, PR-AUC = 0.757, and Brier Score = 0.223. This represents a + 16% improvement over the DoomBot benchmark (F1 = 0.647) and +24.6% over static GNNs like GraphSAGE (F1 = 0.602). The consistency of results across threshold settings (τ=0.90–1.00), with F1-scores ranging from 0.730 to 0.771 and MCC reaching 0.488, along with well-calibrated probability estimates (Brier Scores: 0.176–0.223), demonstrate methodological robustness.

**Explainability at Scale**: GNNExplainer validation (Fidelity+ = 0.827, Characterization = 0.913) confirms that identified features genuinely drive model predictions. The analysis reveals consistent importance of lagged productivity, employment, and population variables, while trading partner contributions highlight the USA (22.6%) and China (16.5%) as dominant network influences. Unlike SHAP-based explanations for shallow methods, which remain fixed across countries, GNNExplainer provides country-customized interpretability that captures each nation’s unique position in the trade network. This transparency supports actionable decision-making for policymakers and stakeholders.

**Dynamic Modeling of Global Trade**: By representing economies as Discrete-Time Dynamic Graphs (DTDGs), **EconoGNN** effectively captures both temporal and topological shifts in global economic networks. This approach enables granular insights into crisis propagation and recovery patterns, addressing critical issues in dynamic economic modeling.

Through its comprehensive approach, **EconoGNN** serves as both a research tool and a practical resource for policymakers and international organizations. By identifying drivers of resilience and vulnerability, the framework supports the design of strategies to mitigate economic shocks and foster sustainable growth.

In future work, **EconoGNN** could be extended to integrate additional data sources, refine model architectures, and explore applications in domains like climate resilience and global health crises. This study highlights the transformative potential of graph-based machine learning in addressing complex, multi-dimensional challenges in global economics. By advancing toward real-time scalability, EconoGNN could evolve into a dynamic monitoring tool, capturing how structural shocks diffuse across economies and industries as they unfold.

## Supporting information

**S1 Code. EconoGNN framework reproduction package.**

All data processing scripts, labeling algorithms, and model configurations used in this study. Available at: https://github.com/AraujoMarcus/ECONO_GNN. Archived version: https://doi.org/10.5281/zenodo.18751102

**S1 Sources. External data sources.**

The raw datasets used in this study are publicly available from the following sources: United Nations COMTRADE database (https://comtradeplus.un.org/TradeFlow), Penn World Table (https://www.rug.nl/ggdc/productivity/pwt/?lang=en), and Resource Trade Earth (https://resourcetrade.earth/).
